# EPDR1 levels and tumor budding predict and affect the prognosis of bladder carcinoma

**DOI:** 10.3389/fonc.2022.986006

**Published:** 2022-10-07

**Authors:** Yue Yang, Hong Xu, Han Zhu, Dan Yuan, Hanchao Zhang, Zhengdao Liu, Faliang Zhao, Guobiao Liang

**Affiliations:** ^1^ Department of Urology, Affiliated Hospital of Zunyi Medical University, Zunyi, China; ^2^ Department of Urology, Affiliated Hospital and Clinical Medical College of Chengdu University, Chengdu, China; ^3^ Medical College of Soochow University, Suzhou, China; ^4^ Department of Pathology, Affiliated Hospital of Zunyi Medical University, Zunyi, China

**Keywords:** biomarker, EPDR1, bladder cancer, prognosis, tumor margin

## Abstract

**Background:**

Bladder carcinoma is a common malignancy of the urinary system. The previous study showed that EPDR1 expression was significantly related to the carcinogenesis and progression of bladder carcinoma

**Methods:**

We retrospectively reviewed the records of 621 patients who were newly diagnosed with bladder carcinoma between January 2018 and August 2020 at The Affiliated Hospital of Zunyi Medical University. We conducted immunohistochemistry of EPDR1 in tumor tissues. Meanwhile, tumor budding evaluation was also carried out by 2 independent experienced pathologists.

**Results:**

80 patients were included in this study with a median age of 66 years (range; 42–88 years). 45% of the patients (36/80) were non-muscle-invasive bladder carcinoma patients, while 55% of muscle-invasive bladder carcinoma(44/80). The follow-up time was from 6 months to 36 months. We found that there were significant differences in expression of EPDR1 in the tumor pT stages(p<0.05), pM stages(p<0.05), and pN stages(p<0.05). Meanwhile, a higher expression of EPDR1 indicated a worse outcome for the patient(p<0.05). A tendency toward a worse status of the patient was accompanied by a high positive rate (p<0.001). Moreover, the IOD of EPDR1 had a positive relationship with TB (p<0.05). Furthermore, we found that EPDR1 and tumor budding could be crucial factors for affecting the prognosis of bladder carcinoma, even better than pTMN(Riskscore=(0.724)* pT_stage +(4.960) *EPDR1+(4.312)*TB).

**Conclusion:**

In conclusion, bladder cancer patients with higher expression levels of EPDR1 had worse survival outcomes. The combination of TB and EPDR1 levels could predict the prognosis for muscle-invasive bladder cancer patients.

## Introduction

Bladder carcinoma is a common malignancy of the urinary system and is the tenth most common neoplasm in the world (1). According to the latest cancer database (2), approximately 440,000 new cases are confirmed to be bladder carcinoma each year, and approximately 160,000 patients die from bladder carcinoma each year. These numbers have been rapidly increasing over the years. Bladder carcinoma can be divided into two categories according to the pathological types: nonmuscle-invasive bladder carcinoma (NMIBC, approximately 75%) and muscle-invasive bladder carcinoma (MIBC, approximately 15%) (3). Moreover, bladder tumors easily progress and metastasize. Thus, NMIBC can easily progress into MIBC, which is the main cause of bladder carcinoma deaths. However, there is a lack of specific biomarkers that can predict tumor behaviors and the prognosis of bladder carcinomas.

The ependymin-related 1 (EPDR1) gene, also known as MERP1 and UCC1, was proven to be associated with a variety of tumors (4). In our previous study, we found that EPDR1 expression was significantly related to different grades and metastases in bladder carcinoma patients. Additionally, it might play a role in affecting the potential molecular mechanisms involved in the carcinogenesis and progression of bladder cancer (5). Therefore, we collected a large number of bladder carcinoma tumor tissues to explore and confirm the significance of EPDR1 in bladder carcinoma. Moreover, we used “tumor budding”, an emerging prognostic biomarker in solid cancers, to assist EPDR1 in predicting the prognosis of bladder carcinoma.

Tumor budding (or “sprouting”, TB) is usually defined as an isolated single cancer cell or small groups of up to 5 tumor cells that are scattered in the stroma at the invasive tumor cell front (6). It was first introduced in the pathology of tumors by Imai in the 1950s (7), and later, an increasing amount of evidence supported the prognostic value of tumor budding in solid cancers (8, 9). According to the International Tumor Budding Consensus Conference (ITBCC) (10), the tumor budding assessment of colorectal cancer (CRC) was included in a standardized reporting process for pathologists. Recently, Soriano also suggested that TB could be an independent predictive factor for MIBC (11).

In our present study, we aimed to explore the differential expression of EPDR1 protein in the pathology of graded bladder carcinoma and found a relationship between various expression levels of EPDR1 and TB and the prognosis of MIBC patients. We aimed to build a prediction model using the pathological results for EPDR1 and TB to predict the prognosis of bladder carcinoma patients, thus providing a reference for clinicians.

## Methods and materials

### Patients

We retrospectively reviewed the records of 621 patients who were newly diagnosed with bladder carcinoma between January 2018 and August 2020 at The Affiliated Hospital of Zunyi Medical University. All patients underwent partial or radical cystectomy. The final pathology confirmed the type of bladder cancer, and only urothelial cancer was included in this study. All the clinical data of patients were collected, and patients with less than 6 months of follow-up were excluded. Therefore, 80 cases were included in the analysis. This study was conducted according to the guidelines of the Declaration of Helsinki and approved by the Affiliated Hospital of Zunyi Medical University ethical committee. All the patients agreed to participate in this study.

### Immunohistochemistry of EPDR1

We collected 80 tumor tissues from the included patients, and these tissues were formalin-fixed and paraffin-embedded. Then, tumor samples were sectioned at 2 mm thickness and mounted on coated glass slides. After incubation in oven at 60°C for 30 min, heat-induced antigen retrieval was performed. The tissues were immersed in xylene and a descending series of graded ethanol concentrations for deparaffinization and rehydration. Endogenous peroxidase activity was blocked by H2O2, and antigen complexes were visualized by an Envision Flex Kit combined with goat serum. Antibodies against EPDR1 (1:80 dilution) were applied, and haematoxylin and eosin (HE) staining was carried out on all slides.

The staining results were examined by two experienced pathologists (Yuan Dan and Hanchao Zhang) with a microscope, and the final results were calculated by immunohistochemical image greyscale analysis of the integral optical density (IOD). Image-Pro Plus software was used to digitally quantify the percent area stained relative to the total plaque area and staining intensity. First, a micron-scale bar was added to the images to calibrate the Image-Pro Plus software before quantitative analyses. Then, we opened the immunohistochemical staining pictures that needed to be measured and converted the grey value to the optical density value in Image-Pro Plus software. The measurement parameters were set as the integrated optical density value (IOD). Furthermore, we circled the area to be measured, set the color selection, and calculated the IOD in the area. Finally, three sections were taken from each specimen to calculate the mean IOD value as the semiquantitative expression of EPDR1. The positive localization and semiquantitative expression of EPDR1 in each group were observed.

### Tumor budding evaluation

Only muscle-invasive urothelial carcinoma patients (pT3 or pT4) who underwent radical cystectomy were included in this analysis. Based on HE staining, tumor budding was defined as an isolated single cancer cell or small groups of up to 5 tumor cells scattered in the stroma at the invasive tumor cell front. Low magnification (X100) was used to visualizing the entire tumor invasion front, and after finding the area with the highest TB density, TB was counted under high magnification (X200). Two independent experienced pathologists (Yuan Dan and Hanchao Zhang) carried out the quantification of TB. If the number of TB was more than 6, it was defined as high expression.

### Statistical Analysis

STATA (version 13.1.) was used for the statistical analysis. Kaplan‒Meier curves and ROC curves were used to estimate survival. To explore the associations between EPDR1 and TB, chi-square (x2) and Mann‒Whitney U tests were used. Using a combination of certain vital clinicopathological covariates, such as age, pTMN, and metastasis, multivariate analysis was performed to verify the potential role of “EPDR1” in bladder cancer patients. All statistical tests are presented with a p value and estimates with a confidence interval of 95%. P <0.05 was considered statistically significant. The 3-year disease-free survival (DFS) and overall survival (OS) of the patients (n=80) were observed.

## Results

### Patient clinicopathological data

Eighty patients were included in this study, and the median age was 66 years (range: 42–88 years). Forty-two females and 38 males were diagnosed with urothelial carcinoma of the bladder. Forty-five percent of the patients (36/80) had nonmuscle-invasive bladder cancer, and 55% had muscle-invasive bladder carcinoma (44/80). The follow-up time ranged from 6 months to 36 months. The full basic characteristics of the included patients with bladder carcinoma are presented in **Table 1**.

**Table 1:** Basic characteristics of included patients with bladder carcinoma.

	Chararters	Number
	Patients	80
Age (year)	Mean (SD)	66.3 (11.6)
	Median [Min, max]	66 [42,88]
Gender	Female	42
	Male	38
pT_stage	T1	26
	T2	10
	T3	28
	T4	16
pN_stage	N0	70
	N1	6
	Nx	4
pM_stage	M0	64
	M1	10
	Mx	6
Grade	Low	32
	High	48
Invasiveness	Yes	32
	Not	48
Tumor size(cm)	Mean (SD)	2.65(1.98)
Survival statistics (3 years)	Disease-free survival (DFS)	37.50%
	Overall survival (OS)	76.25%

### Expression of EPDR1

**Figure 1:**
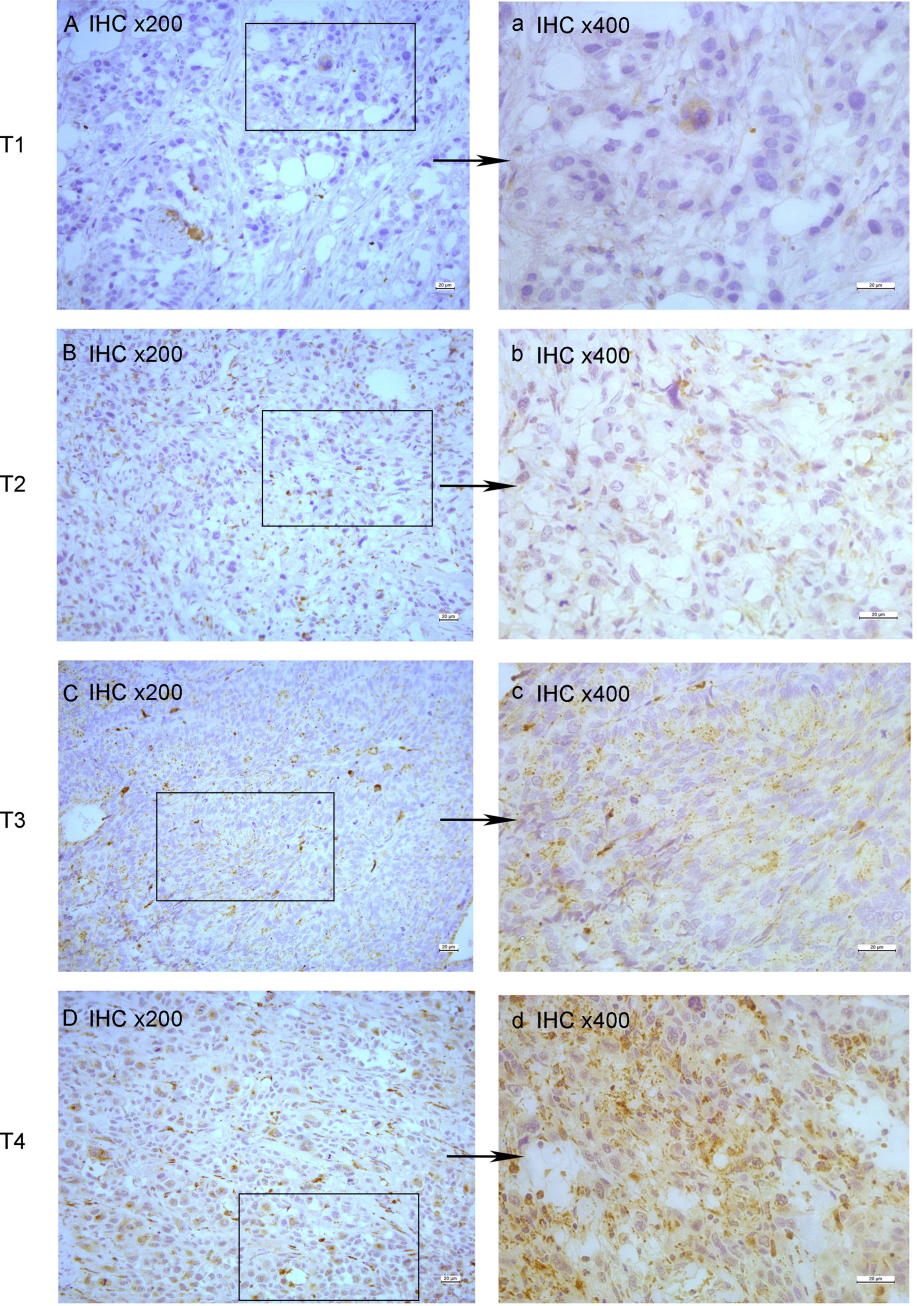
Immunohistochemistry(IHC) of EPDR1 in different pT-stage of bladder cancer. stage: T1, **(A)** IHC x200 and a: IHC x400; stage: T2, **(B)** IHC x200 and b: IHC x400; stage: T3, **(C)** IHC x200 and c: IHC x400; stage: T4, **(D)** IHC x200 and d: IHC x400.

**Figure 2:**
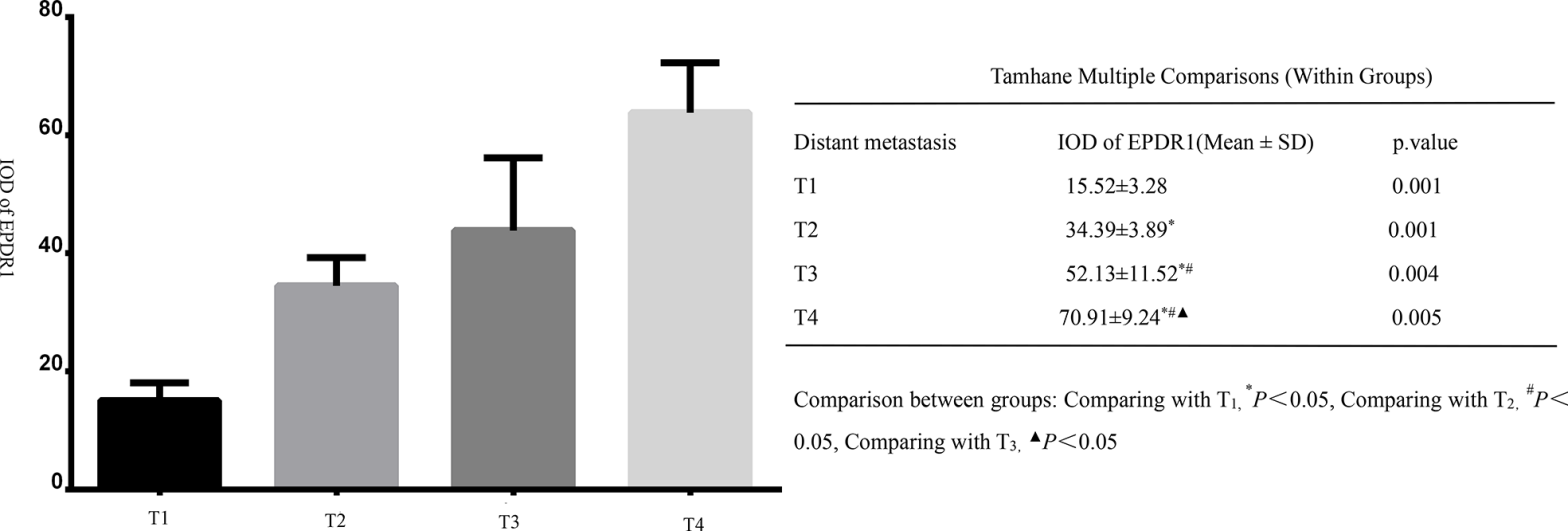
Integral optical density(IOD) of Immunohistochemistry (EPDR1) in different clinical tumor stages(pT stage) of bladder cancer. Gragh: mean IOD of EPDR1 in different pT stages(left p<0.05); table: Tamhane multiple comparisons within Groups of different pT stages of bladder cancer(right: pT1 stage, EPDR1 15.52 ± 3.28, p=0.001; pT2 stage, EPDR1 34.39 ± 3.89, p=0.001; pT3 stage, EPDR1 52.13 ± 11.52, p=0.004; pT4 stage, EPDR1 70.91 ± 9.24, p=0.005).

**Figure 3:**
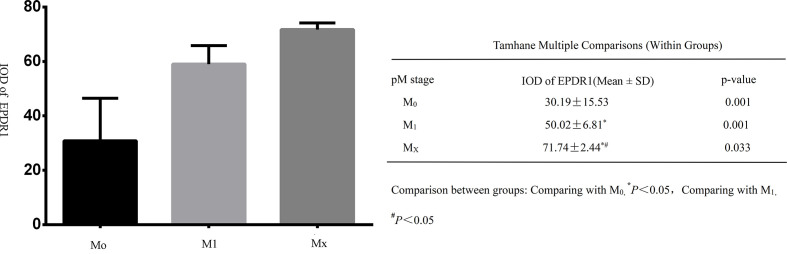
Integral optical density(IOD) of Immunohistochemistry (EPDR1) in different clinical metastasis stages(pM stage) of bladder cancer. Gragh: mean IOD of EPDR1 in different pM stages(left p<0.05); table: Tamhane multiple comparisons within Groups of different pM stages of bladder cancer(right: M0, EPDR1 30.19 ± 15.53, p=0.001; M1, EPDR1 50.02 ± 6.81, p=0.001; Mx, EPDR1 70.74 ± 2.44, p=0.033).

**Figure 4:**
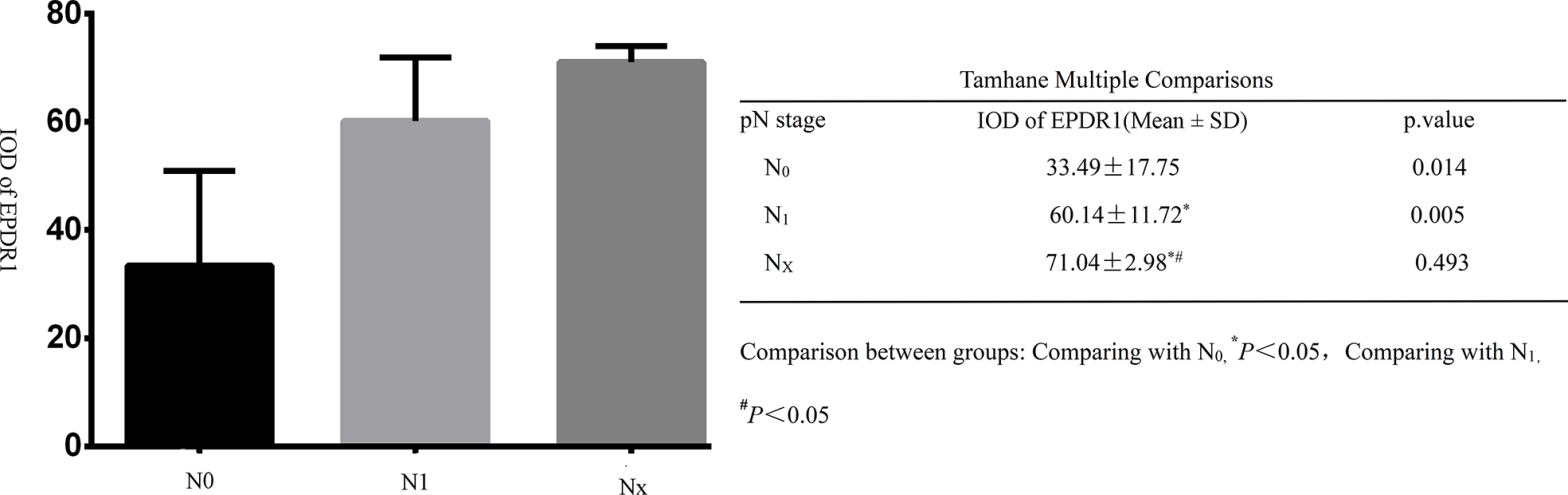
Integral optical density(IOD) of Immunohistochemistry (EPDR1) in different clinical lyphm node stages(pN stage) of bladder cancer. Gragh: mean IOD of EPDR1 in different pN stages(left p<0.05); table: Tamhane multiple comparisons within Groups of different pN stages of bladder cancer(right: N0, EPDR1 33.49 ± 17.75, p=0.014; N1, EPDR1 60.14 ± 11.72, p=0.005; Nx, EPDR1 71.04 ± 2.98, p=0.493).

All tumor tissues were subjected to immunohistochemistry for EPDR1 (**Figure 1**). We used immunohistochemical image greyscale analysis to calculate the integral optical density (IOD) of EPDR1 (semiquantitative analysis). We found significant differences in the IOD of EPDR1 in the tumor pT stage(pT1 stage, EPDR1 15.52 ± 3.28, p=0.001; pT2 stage, EPDR1 34.39 ± 3.89, p=0.001; pT3 stage, EPDR1 52.13 ± 11.52, p=0.004; pT4 stage, EPDR1 70.91 ± 9.24, p=0.005; **Figure 2**), pM stage(M0, EPDR1 30.19 ± 15.53, p=0.001; M1, EPDR1 50.02 ± 6.81, p=0.001; Mx, EPDR1 70.74 ± 2.44, p=0.033; **Figure 3**), and pN stage(N0, EPDR1 33.49 ± 17.75, p=0.014; N1, EPDR1 60.14 ± 11.72, p=0.005; Nx, EPDR1 71.04 ± 2.98, p=0.493; **Figure 4**). Notably, there was no significant difference in EPDR1 expression between the N1 and Nx stages (p=0.493).

### Tumor budding

**Figure 5:**
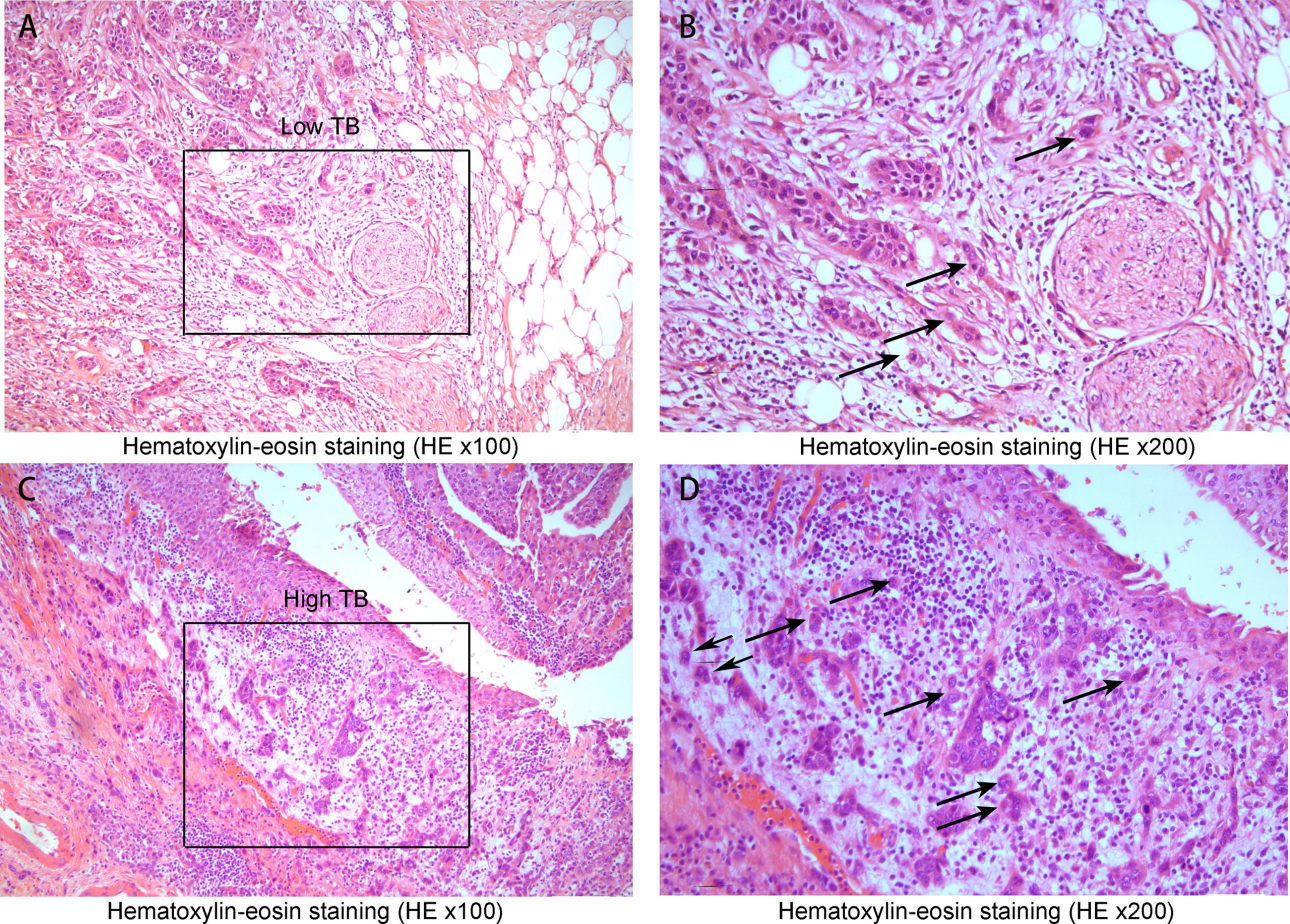
Hematoxylin-eosin staining(HE x100, x200). **(A)** A low tumor budding case(Low TB x100) and **(B)** low tumor budding case (the black square lesion in **(A)** was set to x200). The black circle indicates a cluster of cancer cells inside the circle where TB was less than five. **(C)** A high tumor budding case (x100) and **(D)** a high tumor budding case (the black square lesion in **(C)** was set to x200). There were 6 or more budding foci, which were isolated single cancer cells (allows) or a cluster composed of fewer than five cancer cells (black circle).

**Figure 6:**
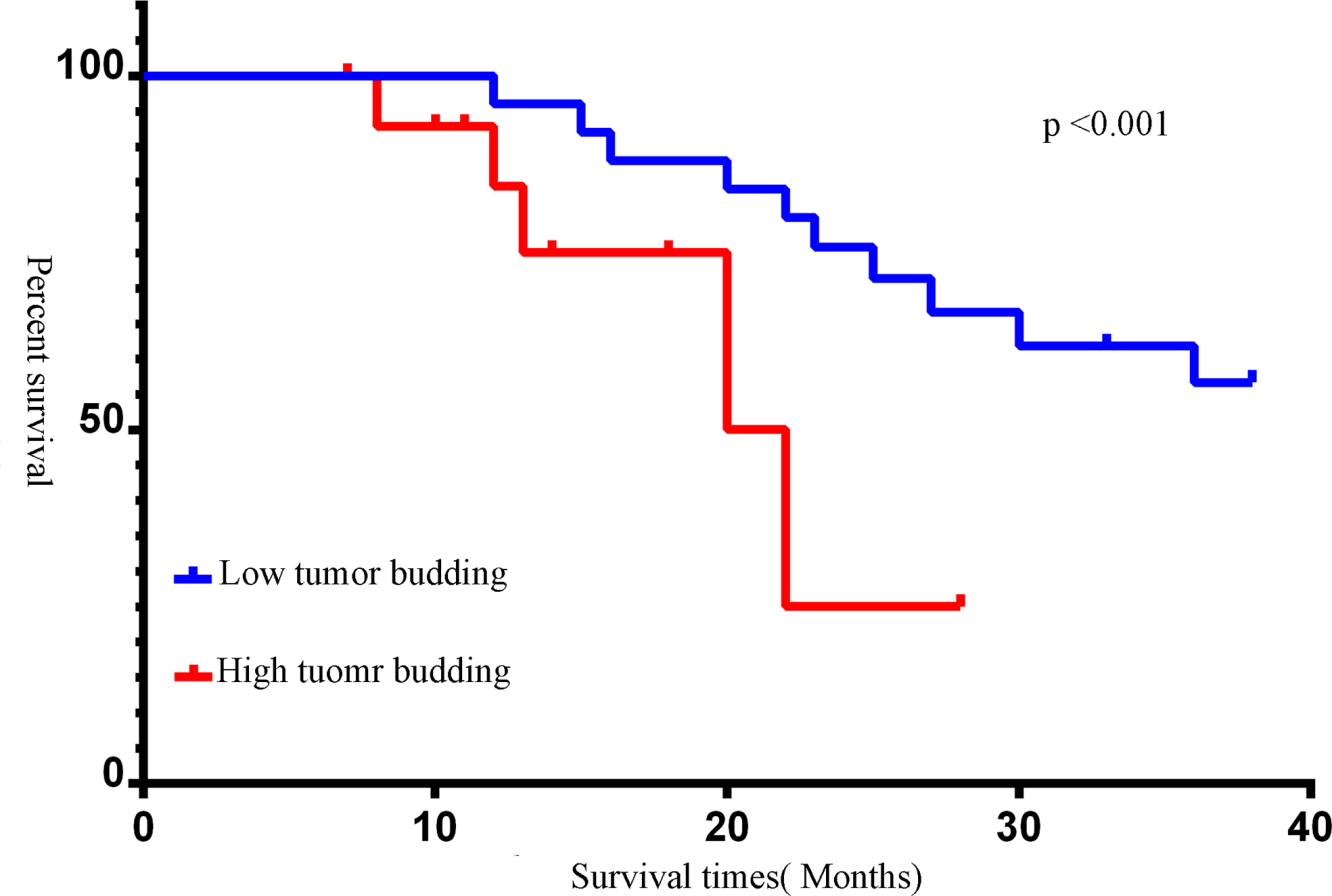
Kaplan-Meier survival curves of different tumor budding(TB). The number of TB was more than 6, we defined it as a high expression. Red line: tumor budding high; blue line: tumor budding low (p<0.05).

**Figure 7:**
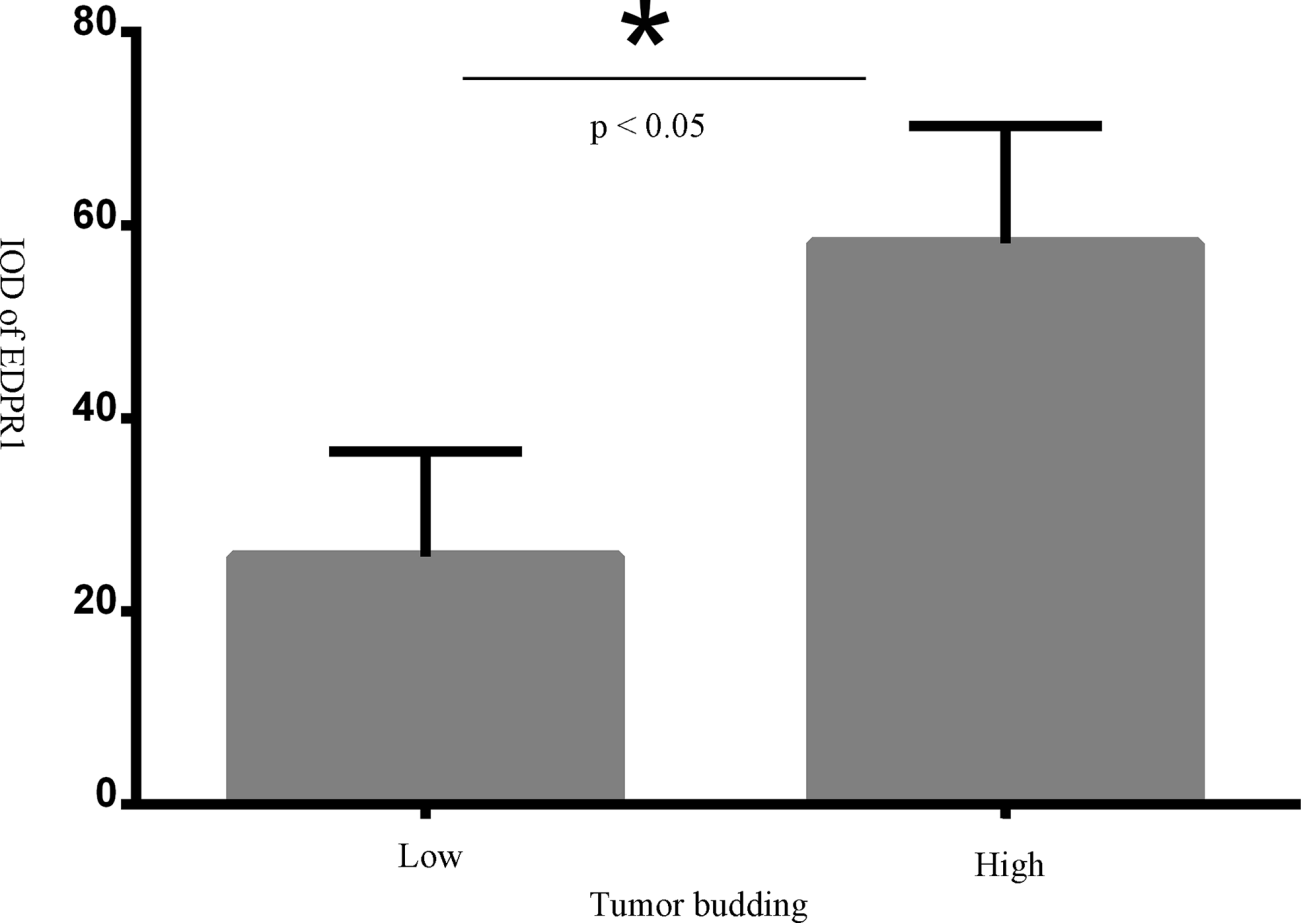
The relationshp between tumor budding(TB) and IOD of EPDR1(p<0.05). * represent a significance of statistic difference.

Forty-four muscle-invasive bladder cancer patients were included in this analysis. Based on haematoxylin and eosin (HE) staining of tumor tissues, 2 independent experienced pathologists evaluated the number of TB cases. We defined a sample as highly positive if the number of TB cases was more than 6 (**Figure 5**). A tendency toward a worse status of the patient was accompanied by a high positive rate (**Figure 6**, p<0.001). Moreover, the IOD of EPDR1 had a positive relationship with TB (**Figure 7**, p<0.05).

### Survival statistics

**Figure 8:**
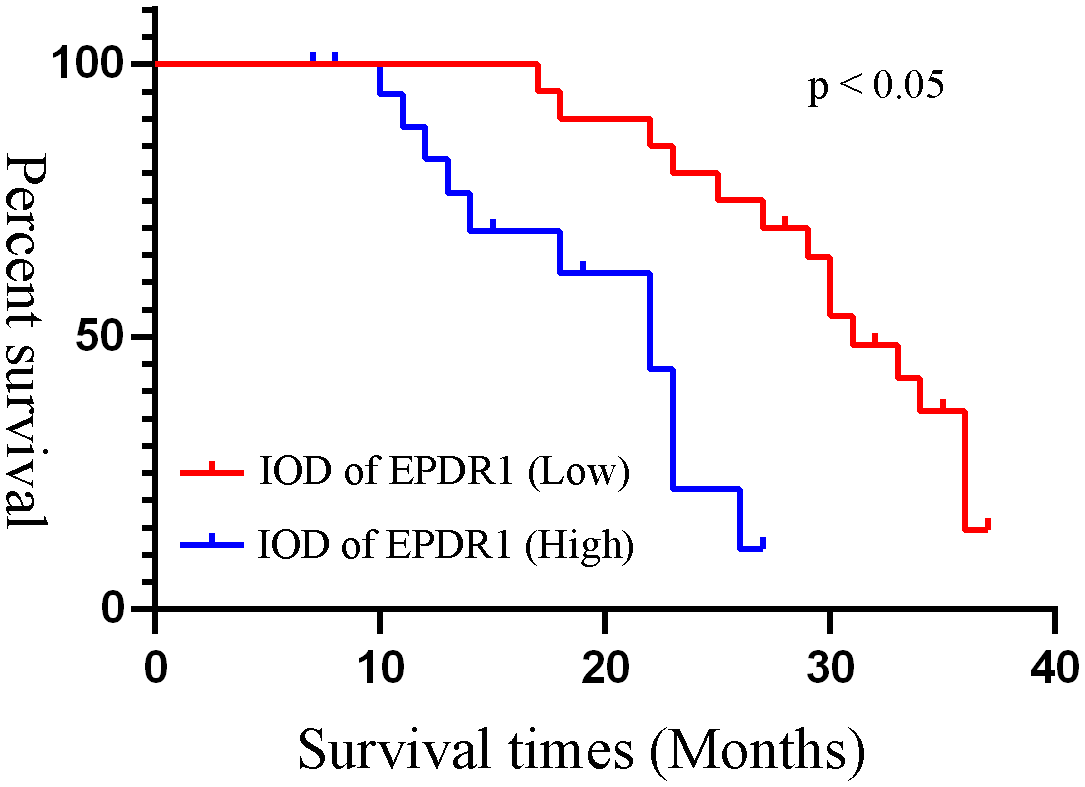
Kaplan-Meier survival curves of different IOD of EPDR1. Based on the median IOD of EPDR1, we divided the patients into two groups as high and low expression of EPDR1. Red line: IOD of EPDR1 low; blue line: IOD of EPDR1 high(p<0.05).

**Figure 9:**
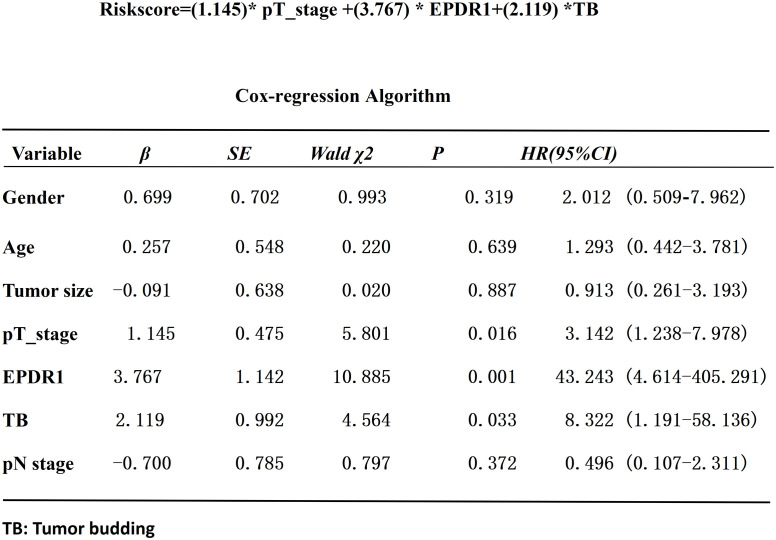
Cox-regression algorithm analysis of the risk factors in bladder cancer patients.EPDR1(p=0.001), TB(p=0.033), and pT(p=0.016) could be crucial factors(Riskscore=(0.724)* pT_stage +(4.960) *EPDR1+(4.312)*TB). * represent multiplication.

Based on the median IOD of EPDR1 (median IOD of EPDR1 = 34.85), we divided the patients into two groups: high and low EPDR1 expression. Kaplan‒Meier survival curves were generated to determine the relationship between EPDR1 and the survival rate of bladder cancer patients. We found that high expression of EPDR1 indicated a poor outcome (**Figure 8**, p<0.05). Using a combination of certain clinical and pathological covariates, such as age, sex, pTNM, tumor size, and TB, a Cox regression algorithm was conducted to explore the risk factors and build a predictive model for bladder cancer patients(Riskscore=(0.724)* pT_stage +(4.960) *EPDR1+(4.312)*TB, **Figure 9**). We found that EPDR1 (p=0.001), TB (p=0.033), and pT (p=0.016) could be crucial factors affecting the prognosis of bladder carcinoma.

## Discussion

Bladder cancer causes approximately 200,000 deaths per year worldwide (12), and it has different disease-specific mortality rates for noninvasive tumors and invasive tumors. The most common and effective treatment for bladder cancer is surgery, but over 50% of muscle-invasive tumors recur within 2 years after cystectomy (13). With higher grades and more invasive BLCA, patients are more likely to experience widespread metastasis and cancer death (14). However, the lack of specific treatments and predictions for most advanced bladder cancers is a challenge for urologists.

In our study, we found that the expression of EPDR1 in tumor tissues was significantly associated with the grade, metastasis, invasion, and survival of bladder cancer. These results were consistent with our previous study (5). EPDR1 was highly expressed in bladder cancer patients with high tumor stages (pT), wider metastases, and positive lymph nodes, all of which indicate a worse prognosis. Similarly, a study by F. Gimeno-Valiente showed that the expression of EPDR1 was upregulated in 101 colorectal cancer (CRC) patients in a prospective cohort, and they found that a high level of EPDR1 expression is associated with T and M parameters in CRC (15). Their research also supported the inclusion of EPDR1 in gene panels that can be used to improve the molecular subtyping of CRC. In several other works in the literature (16–19), the gene expression profile of EPDR1 was also elevated in other cancers, such as hepatocellular carcinoma, pancreatic adenocarcinoma, and stomach adenocarcinoma. Our previous study found that EPDR1 was closely associated with AKT1, PIK3CA, and FGFR3 (5). Previous studies (13, 19) have demonstrated that PTEN/PI3K/AKT/mTOR signalling pathways participate in bladder carcinogenesis. Thus, we speculated that EPDR1 could affect the progression and metastasis of bladder cancer. Ultimately, EPDR1 may be an actionable target in bladder cancer. In this study, we focused on the pathological features of EPDR1 and tumor budding and further investigated the potential clinical prognosis of EPDR1 and the survival of bladder cancer patients. Finally, we attempted to quantify the risk of differential EPDR1 expression and tumor budding with a predictive model for bladder cancer patients.

Considering the lower invasiveness and better prognosis of NMIBC compared with MIBC, tumor budding was infrequently observed in the pathological specimens of NMIBC (20). Therefore, our study explored tumor budding in MIBC. In our study, a high level of positive expression of tumor budding indicated poor survival; thus, we suggest that tumor budding is a parameter for worse overall survival in muscle-invasive bladder cancer (MIBC) when compared with NMIBC. Recently, many studies have also supported the notion that tumor budding is related to bladder cancer outcomes. In a study by Markus Eckstein (20), 92 patients with stage pT1 NMIBC were enrolled, and a higher expression of tumor budding was observed in a more aggressive and invasive stage of pT1 NMIBC with a worse outcome. Nicolas Brieu conducted a study that was comprised of 100 MIBC patients, and their findings showed that tumor budding provided prognostic value for muscle-invasive bladder cancer patients and could even serve a better model than TNM staging (21). Although tumor budding could be a potential important risk factor for bladder cancer, there were certain specifically crucial factors, such as pTMN stages, that predicted the prognosis of bladder cancer patients. A combination of more clinical and pathological covariates could help urologists have a better understanding and comprehensive assessment of the prognosis of bladder cancer patients.

In this study, we found that bladder cancer patients had shorter survival rates, and higher expression of EPDR1 and higher positivity for TB were detected in MIBC tumor tissues when compared with NMIBC. Previous studies (12, 13) had already demonstrated that MIBC were more aggressive than NMIBC, thus MIBC could easily progress to distant metastasis. To a certain extent, our study showed that EPDR1 and TB could represent the invasiveness of the bladder tumor cells. Moreover, we also demonstrated that the expression of EPDR1 had a positive relationship with TB (p<0.005). Combining several factors, we built an ideal prediction model for the prognosis of muscle-invasive bladder cancer patients (Riskscore=(0.724)* pT_stage +(4.960) *EPDR1+(4.312)*TB, **Figure 9**), and EPDR1 (p=0.001), TB (p=0.033), and pT (p=0.016) could be crucial factors. The results of the analysis even suggested that EPDR1 and TB could be more valuable for the prognosis of muscle-invasive bladder cancer patients than TNM staging.

In conclusion, bladder cancer patients with higher expression levels of EPDR1 had worse survival outcomes. The combination of TB and EPDR1 levels could predict the prognosis for muscle-invasive bladder cancer patients.

## Data availability statement

The original contributions presented in the study are included in the article. Further inquiries can be directed to the corresponding author.

## Ethics statement

The studies involving human participants were reviewed and approved by affiliated hospital of Zunyi Medical university ethical committee (KLL-2021-300). The patients/participants provided their written informed consent to participate in this study.

## Author contributions

Methodology, writing-original draft preparation, YY. Software, HX and HZ. Formal analysis, DY and ZL. Data curation, FZ and HCZ. Writing-review and editing, GL. All authors contributed to the article and approved the submitted version.

## Funding

Special fund for Training outstanding Young Scientific and technological Talents of Guizhou Province, Grant Number 2015 (31), Project of Affiliated Hospital of Chengdu University, Grant Number Y202224.

## Acknowledgments

Thanks for the project: “Young Crops of Talents” of The Affiliated Hospital of Chengdu University.

## Conflict of interest

The authors declare that the research was conducted in the absence of any commercial or financial relationships that could be construed as a potential conflict of interest.

## Publisher’s note

All claims expressed in this article are solely those of the authors and do not necessarily represent those of their affiliated organizations, or those of the publisher, the editors and the reviewers. Any product that may be evaluated in this article, or claim that may be made by its manufacturer, is not guaranteed or endorsed by the publisher.
